# Do all roads lead to resistance? State road density is the main impediment to gene flow in a flagship species inhabiting a severely fragmented anthropogenic landscape

**DOI:** 10.1002/ece3.7635

**Published:** 2021-05-06

**Authors:** Katharina Westekemper, Annika Tiesmeyer, Katharina Steyer, Carsten Nowak, Johannes Signer, Niko Balkenhol

**Affiliations:** ^1^ Wildlife Sciences University of Goettingen Goettingen Germany; ^2^ Conservation Genetics Section Senckenberg Research Institute and Natural History Museum Frankfurt Gelnhausen Germany; ^3^ Department of Ecology and Evolution Johann Wolfgang Goethe‐University, Biologicum Frankfurt am Main Germany

**Keywords:** barrier, circuit theory, commonality analysis, connectivity, European wildcat, fragmentation, gene flow, landscape genetics, resistance

## Abstract

**Aim:**

Connectivity conservation is ideally based on empirical information on how landscape heterogeneity influences species‐specific movement and gene flow. Here, we present the first large‐scale evaluation of landscape impacts on genetic connectivity in the European wildcat (*Felis silvestris*), a flagship and umbrella species for connectivity conservation across Europe.

**Location:**

The study was carried out in the core area of the distributional range of wildcats in Germany, covering about 186,000 km^2^ of a densely populated and highly fragmented landscape.

**Methods:**

We used data of 975 wildcats genotyped at 14 microsatellites and an individual‐based landscape genetic framework to assess the importance of twelve landscape variables for explaining observed genetic connectivity. For this, we optimized landscape resistance surfaces for all variables and compared their relative impacts using multiple regression on distance matrices and commonality analysis.

**Results:**

Genetic connectivity was best explained by a synergistic combination of six landscape variables and isolation by distance. Of these variables, road density had by far the strongest individual impact followed by synergistic effects of agricultural lands and settlements. Subsequent analyses involving different road types revealed that the strong effect of road density was largely due to state roads, while highways and federal roads had a much smaller, and county roads only a negligible impact.

**Main conclusions:**

Our results highlight that landscape‐wide genetic connectivity in wildcats across Germany is strongly shaped by the density of roads and in particular state roads, with higher densities providing larger resistance to successful dispersal. These findings have important implications for conservation planning, as measures to mitigate fragmentation effects of roads (e.g., over‐ or underpasses) often focus on large, federally managed transportation infrastructures. While these major roads exert local barrier effects, other road types can be more influential on overall connectivity, as they are more abundant and more widespread across the landscape.

## Graphical abstract

The study presents the first large‐scale investigation on connectivity in the European wildcat across its entire distributional range in Germany, where the endangered species serves as an important flagship species for national and local defragmentation efforts. Using genetic data of 975 individuals, we were able to identify five different landscape variables that significantly affect realized gene flow in the species; road density was by far the most influential of these variables. Specifically, we found that overall connectivity of wildcat in Germany is strongly influenced by state roads.

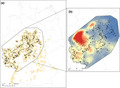

## 1 INTRODUCTION

Landscape fragmentation and habitat loss continue to threaten biodiversity around the globe (Crooks et al., 2017; Haddad et al., 2015; Tscharntke et al., 2012). Numerous studies have documented negative consequences on distribution and abundance, as well as on genetic variation and physiological processes (Fahrig, 2003; Fischer & Lindenmayer, 2007; Frankham, 2005; Lino et al., 2019; Schlaepfer et al., 2018). To prevent the further loss of species, their genetic diversity, and ecosystem functions, conservation efforts increasingly aim to maintain or improve landscape connectivity (Hilty et al., 2019; Keeley et al., 2019). Sufficient connectivity of a landscape allows for dispersal movements and genetic exchange among remaining populations, thereby increasing individual fitness, population viability, and species persistence.

Approaches for assessing landscape effects on connectivity often use the concept of landscape resistance, which represents the willingness or ability of an organism to move through a particular environment (Zeller et al., 2012). Estimating landscape resistance is typically achieved by parameterizing the relative cost of environmental variables to movement and gene flow from empirical data, with lower resistance values indicating a higher probability of successfully moving through an area (Balkenhol et al., 2020; Zeller et al., 2017). Thus, connectivity planning based on landscape resistance should use actual data on gene flow or movement and such data should ideally be available for multiple, conservation relevant or umbrella species (Diniz et al., 2018; Meurant et al., 2018).

Habitat fragmentation can have particularly negative effects in densely populated regions with abundant traffic infrastructures. Germany, for instance, is among the most fragmented countries in Europe and characterized by a dense human population (233 inhabitants/km^2^), large areas used for settlements and traffic infrastructures (ca. 51,000 km^2^ or ca. 14% of the total land area), and an extensive road network (2.5 km/km^2^) with heavy vehicle traffic (Federal Ministry of Transport and Digital Infrastructure (BMVI), 2019; Federal Statistical Office of Germany (Destatis), 2019). The German federal government is committed to improve landscape connectivity across the country, and environmental agencies collaborate with various local and national nongovernmental organizations on different connectivity initiatives (Federal Ministry for the Environment, 2012; Herrmann et al., 2007; Mölich & Vogel, 2018).

As it is typical for many conservation programs, efforts in Germany often focus on charismatic flagship species to generate enough public interest, political support, and financial contributions to make conservation happen on the ground (Caro, 2010). One such species is the European wildcat (*Felis silvestris*), which serves as a highly protected flagship and umbrella species for nature conservation and land‐use planning in many European countries (Gil‐Sanchez et al., 2020; Mattucci et al., 2016; Say et al., 2012). Its habitat use and requirements are well‐studied (Götz et al., 2018; Jerosch et al., 2018; Klar et al., 2008), and several studies already highlighted the importance of functionally connected habitats for the wildcat (Hupe & Simon, 2007; Klar et al., 2012; Mattucci et al., 2016).

In Germany, various local and nationwide conservation projects focus on connecting suitable habitat patches for the wildcat via stepping stones and corridors (Herrmann et al., 2007; Klar et al., 2012; Mölich & Vogel, 2018; Vogel et al., 2009). These projects are based on empirical data, usually derived from local habitat selection studies involving radio‐collared individuals (Klar et al., 2008). While such telemetry data are ideal to determine habitat influences on fine‐scale movement patterns, genetic data are more suitable to detect whether movement also results in actual gene flow across large spatial extents. This is why genetic approaches are increasingly used to estimate species‐specific landscape resistances (Spear et al., 2015; Zeller et al., 2012). Hence, we chose to complement existing knowledge about habitat impacts on European wildcat movement behavior with the first large‐scale assessment of landscape effects on genetic connectivity in the species across Germany. We used genetic data from 975 wildcat individuals distributed across the core range in Germany and employed a multivariate landscape genetics framework to quantify resistance to gene flow provided by different landscape variables, and to compare their relative importance for explaining genetic connectivity.

Our results show that genetic structure across our study region is influenced by a variety of landscape variables, including topographic slope, Continuous Low Traffic Areas, human settlements, forest, agricultural land, and roads. However, road density was by far the most influential variable, with state road densities having the most negative effect on genetic connectivity. These findings improve our understanding of functional landscape connectivity in wildcats and can have important implications for connectivity conservation in Germany and other countries as it moves the focus from large federal roads to the much more abundant state roads with landscape‐wide, rather than just local impacts on gene flow.

## 2 METHODS

### 2.1 Study area and species

**FIGURE 1:**
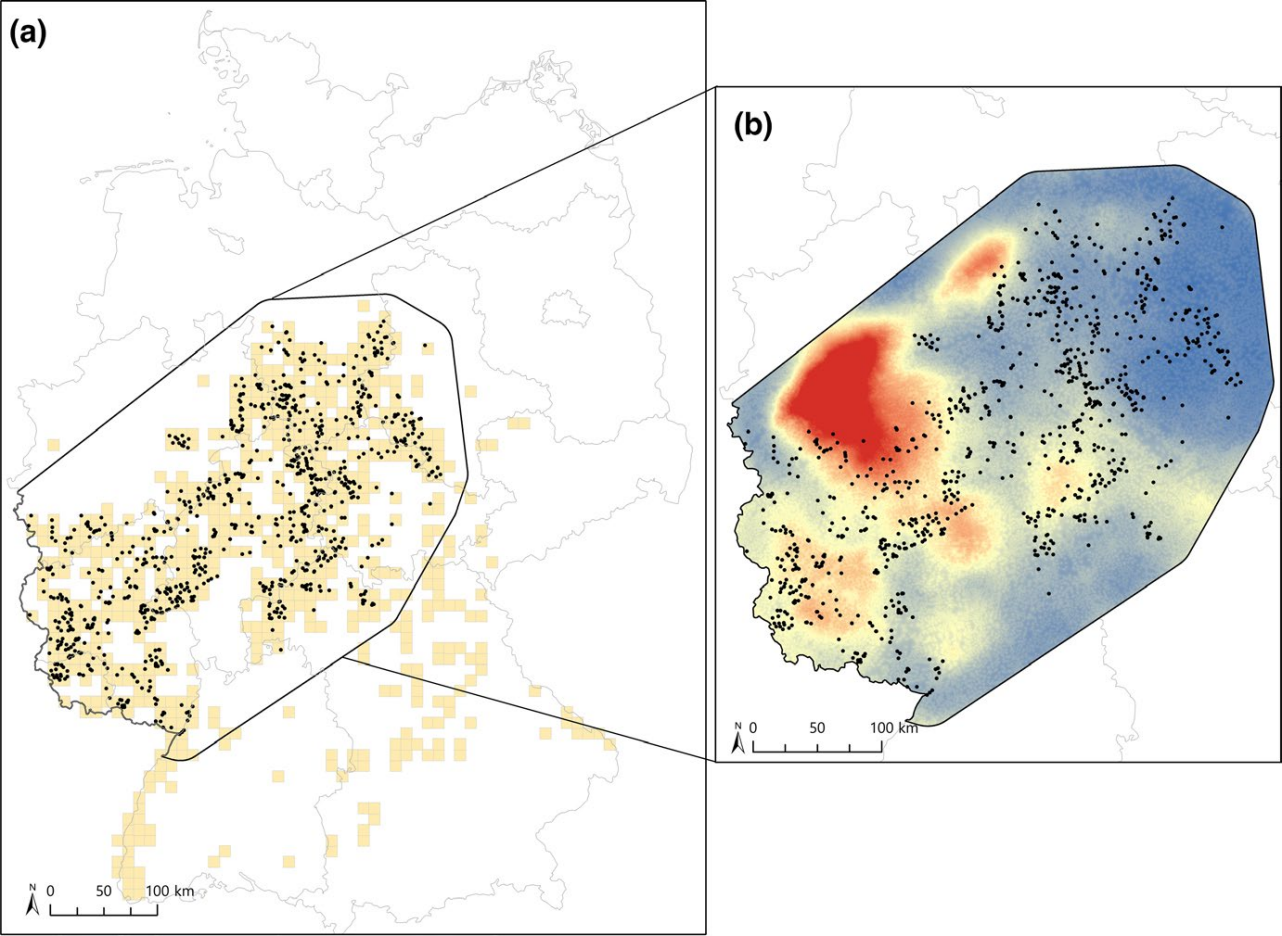
Distribution of wildcat in Germany and position of 975 genetic samples used for this study (a; beige = wildcat data from Balzer et al., 2018, black = genetic samples). Final, multivariate resistance to gene flow within the study area, as inferred from our analyses (b). We weighted each of the resistance surfaces of the selected landscape variables (i.e., road density within a 35 km radius, proportion of forest within a 35 km radius, proportion of forest within a 35 km radius, distance to settlements, distance to Continuous Low Traffic Areas, topographic slope, and straight‐line distance) by its beta weights from commonality analyses and then summed up the resulting layers. The gradient runs from red (high resistance) to blue (low resistance)

Our study took place in the core range of the European wildcat in Germany (Figure 1), covering an area of ca. 186.000 km^2^. The climate is temperate, and the elevation in the study area ranged from 0 to 1,141 m. The wildcat population in Germany is currently estimated between 6,000 and 15,000 individuals (Federal Agency for Nature Conservation (BfN), 2019), and the European wildcat serves as an umbrella species due to its diverse habitat demands. Often described as a forest‐depending species, it requires a diverse habitat of structurally complex forest and meadows. In addition to forest, open land, and near‐natural stream courses, it also uses agricultural fields with stashing crops or copses for foraging (Götz et al., 2018; Jerosch et al., 2017; Klar et al., 2008; Piechocki & Möller, 1983; Streif et al., 2016; Wittmer, 2001). Natural mortality of offspring is high, and the most frequently detected cause of death is road traffic (Echle et al., 2018; Pott‐Dörfer & Raimer, 2007; Simon & Raimer, 2005; Steyer et al., 2016). The European wildcat and the domestic cat can successfully breed with each other (Driscoll et al., 2007). While such hybridization is a major problem in some countries (Pierpaoli et al., 2003), hybridization of wild and domestic cats is rare in Germany (Steyer et al., 2018; Tiesmeyer et al., 2018). The species is strictly protected by the German Federal Nature Conservation Act and included in the Federal Biodiversity Program as a species under special responsibility. It is also listed in Appendix II of the 1979 Bern Convention and in Appendix IV of the Habitats Directive of the European Union.

### 2.2 Genetic data set

We used a subsample of genetic data collected in the frame of long‐term genetic wildcat monitoring in Germany (Steyer et al., 2016; Tiesmeyer et al., 2018). Data were largely based on noninvasively sampled hair, but also included tissue, saliva, feces, and blood samples from different studies. In total, these studies detected 1979 pure‐bred wildcat individuals. Genotyping of all samples was based on 14 microsatellite markers (Menotti‐Raymond et al., 1999; FCA8, FCA88, FCA124, FCA132, FCA149, FCA170, FCA171, FCA232, FCA275, FCA347, FCA364, FCA567, FCA571, and FCA576) and one sex marker (Pilgrim et al., 2005; zinc finger marker). Details of sampling and laboratory protocols can be found in Hartmann et al. (2013) and Steyer et al. (2013). Error rates for allelic dropout and false alleles were calculated as described in Steyer et al. (2016). Since we wanted to focus on the core area of the German wildcat distribution without redundant sampling in our landscape genetic study, we subsampled available data by considering the most recent sample and highest quality genotype per individual in case of multiple detections, and selecting no more than four individuals per EU grid cell (10 × 10 km each) in case multiple individuals were detected in the same cell. We also removed samples without a coordinate and samples with an allelic dropout of >0.5, an amplification success of <0.5, and a sampling date before January 2009. This led to our final data set consisting of 975 recent high‐quality wildcat genotypes (578 males, 350 females, and 47 of undetermined sex) that are continuously distributed across the core area (186,000 km^2^, see Figure 1).

We reran basic population genetic analyses conducted by Steyer et al. (2016) and Tiesmeyer et al. (2018) with our reduced data set to ensure that our data set was representative of the complete data set available for Germany. Results show that both genetic diversity and structure of the subsampled data are highly similar to the full data set (see Appendix S1, Table S1).

### 2.3 Landscape genetic analyses

We quantified genetic connectivity across our study area through the proportion of shared alleles (PSA, Bowcock et al., 1994) an individual‐based genetic distance calculated with “adegenet” (Jombart, 2008). We tested for correlation between pairwise genetic distances and effective distances among individuals, which we estimated using circuit theory (McRae et al., 2015). Specifically, we constructed different resistance surfaces for landscape variables potentially influencing gene flow in wildcats, selected the optimal resistance transformation for each variable, and compared variable importance using multivariate commonality analysis. Further, we conducted a *post hoc* analysis of road type effects on gene flow.

#### 2.3.1 Transformation of landscape variables and creation of resistance surfaces

We used digital layers of different landscape variables with a resolution of 100 m in our analyses (Table 1). Transforming raw values of these layers into resistance surfaces required continuous data, and hence, we processed linear features and most landscape variables in two different ways as a first step: We calculated the distance of each cell in the study area to the closest cell with that landscape variable (e.g., the distance to the nearest forest or nearest road), and we estimated the proportion of landscape variables or the density of linear features, respectively, in radii of 5, 10, or 35 km around each cell. These radii were chosen to reflect the home range sizes reported for wildcats in Germany (Dietz et al., 2016), the largest distances crossed by radio‐tracked wildcats between habitat patches (Klar et al., 2012), and the furthest distance between two genetic samples of the same individual.

**TABLE 1:** Landscape data used to create resistance surfaces

Landscape variable	Source	Hypothesized relationship with gene flow
Continuous Low Traffic Areas	Federal Agency for Nature Conservation 2010	+
Agricultural land	OpenStreetMap 2018 (land use = farmland)	+
Forest	Forest type, European Union, Copernicus Land Monitoring Service 2015	+
Forest fragmentation index, proportion of forest edge	Forest type, European Union, Copernicus Land Monitoring Service 2015	+
Forest fragmentation index, proportion of forest interior	Forest type, European Union, Copernicus Land Monitoring Service 2015	+
Grassland	OpenStreetMap 2018 (land use = grass, greenfield, meadow)	+
Habitat suitability model	Klar et al. (2008)	+
Global Urban Footprint	German Aerospace Center 2016	−
Railways	OpenStreetMap 2018 (land use = railway)	−
River	OpenStreetMap 2018 (waterway = river, canal)	−
Road	ESRI Germany, Federal Agency for Cartography and Geodesy; Open Data Portal 2015	−
Settlement	OpenStreetMap 2018 (landuse = residential, industrial, retail)	−
Topographic slope	Digital elevation model, European Union, Copernicus Land Monitoring Service 2012	−

Note

In the third column, a positive sign indicates a hypothesized positive effect of this variable on gene flow in wildcats (i.e., higher values of the variable lead to lower resistance), while a negative sign indicates that the variable was hypothesized to impede gene flow (i.e., higher values of the variable lead to higher resistance).

Habitat suitability and topographic slope were already continuous variables and received no further transformation. Habitat suitability values were derived from a previously published habitat selection model based on radio‐tracking data of wildcats in southwestern Germany (Klar et al., 2008). For the landscape variables agricultural land, forest, grassland, and roads, we used both transformation variants, distance and proportions/densities to calculate resistance surfaces. For the continuous Global Urban Footprint (Esch et al., 2012, 2013), we calculated resistance surfaces based on the three radii mentioned above. To account for potential effects of forest fragmentation, we also calculated proportions of forest edge and forest interior within a radius of 1 km after classifying forest cover into different structural elements following the approach of Riitters et al. (2000) as implemented in the extension “r.forestfrag” for grass gis 7.4 (Neteler et al., 2012). We used both fragmentation measures, because wildcats are considered a forest‐dependent species, so that a high proportion of forest interior could provide low resistance to dispersal movements. On the other hand, wildcats also use edge habitat as hunting ground (Klar et al., 2008), so that forest edges could serve as conduits during dispersal.

For some landscape variables of low densities, we used the distance to the next occupied cell to transform it into resistance surfaces: railroads, rivers, and Continuous Low Traffic Areas (Federal Agency for Nature Conservation (BfN), 2010). Continuous Low Traffic Areas are identified as areas of at least 100 km^2^ that are not dissected by roads with more than 1,000 vehicles per day, railroads, large canals, or settlements. We used distance to human settlements as resistance surface, because density of human settlements is already covered by Global Urban Footprint.

In a second step, we rescaled layers so that their cell values ranged from 0 to 1 in a way that reflected our resistance hypotheses (Table 1, 3^rd^ column). For example, since we hypothesized higher road density to provide higher resistance to wildcat gene flow, cells with highest road density received a value of 1, and cells with lowest road density received a value of 1. Similarly, we assumed that steeper slopes would present higher resistance for wildcat movement and gene flow, as animals should try to follow paths of low physiological cost (e.g., Dunford et al., 2020). For forest and agricultural land, it was the opposite, as we hypothesized that areas with more forest or agriculture would provide less resistance to gene flow. The rescaled layers, all with a resolution of 100 m, were then transformed into actual resistance surfaces with values ranging between 100 (lowest resistance; this is simply the cell size) and 10,000 (highest resistance; 100 times the cell size) using the formula $\mathrm{resistance}=\mathrm{cellsize}*100^{1‐\mathrm{relative}\;\mathrm{landscape}\;\mathrm{value}}$ (Balkenhol et al., 2020; Mateo‐Sánchez et al., 2015a).

In sum, we based our study on 28 resistance surfaces (Appendix S2.1, Table S2).

#### 2.3.2 Calculation of interindividual effective distances and selection of relevant landscape variables

Based on each of the resistance surfaces described above, we estimated effective distances among all 474,825 pairs of individuals using a high‐performance computing cluster and the software gflow (Leonard et al., 2017). We initially conducted analyses separately for each sex, but as the results were similar for males and females, we pooled sexes for final analyses. gflow is a faster version of the commonly used software circuitscape (Shah & McRae, 2008) and estimates pairwise measures of effective distances based on circuit theory (McRae et al., 2008). We then used a multistep selection procedure to identify the effective distances and underlying landscape variables that best explained genetic distances. Specifically, we used simple Mantel tests (Mantel, 1967) to assess whether effective distances were significantly correlated with genetic distances and partial Mantel tests (Smouse et al., 1986) to test for significance after accounting for the effects of isolation by distance (IBD), that is, after partialing out the effects of geographic (i.e., straight‐line) distances. Mantel tests were calculated in r package “ecodist” (Goslee & Urban, 2007) with 999 permutations to assess significance.

For each representation of each landscape variable, we only retained the transformation (e.g., the proportion of the landscape variable within 35 km radius; see Table 2) with largest significant partial Mantel *r*. Among the remaining effective distances, we selected the ones with a minimum significant partial Mantel *r* of 0.1 for further analyses.

**TABLE 2:** Results of simple and partial Mantel tests of effective distances based on best transformation of each landscape variable and used for commonality analyses

Landscape variable	Mantel *r*	*p*	Partial Mantel *r*	*p*
Agricultural land, 35 km	0.144	.001	0.254	.001
Continuous Low Traffic Areas, distance	0.264	.001	0.176	.001
Forest, 35 km	0.048	.022	0.200	.001
Roads, 35 km	0.342	.001	0.269	.001
Settlement, distance	0.315	.001	0.145	.001
Slope	0.296	.001	0.104	.001

Note

For further selection of landscape variables, see text, and for full results, Table S2.

#### 2.3.3 Multivariate statistical analysis

We used the final set of effective distances, as well as straight‐line distance, for a commonality analysis (Newton & Spurrell, 1967) based on multiple regression on distance matrices (MRDM; Lichstein, 2007; Wang, 2013). Commonality analysis separates the effects of variables into different components and is a particularly useful multivariate approach for landscape genetics, because other methods for analyzing pairwise distances often lead to spurious correlations, thus making it difficult to accurately evaluate the relative importance of explanatory variables (Prunier et al., 2015). Furthermore, commonality analysis can help to disentangle whether variables act independently of each other, or have synergistic effects. In essence, commonality analysis indicates the amount of variance in the dependent variable that is explained by an individual explanatory variable, or a set of multiple explanatory variables. Specifically, impacts of the explanatory variables (i.e., the effective distances) on the dependent variable (i.e., the genetic distances) are divided into unique (*U*, the part of the explained variation attributable to an individual explanatory variable), common effects (*C*, the part of the explained variation attributable to at least two explanatory variables together), and total effects (*T*, the sum of common and individual effects). The sum of the contribution to the overall model R^2^ (% total) can be lower than 100%, indicating suppression, or greater than 100%, pointing to synergistic interactions between variables (Nimon, 2010; Prunier et al., 2017). Detailed information on interpreting commonalities is provided in Prunier et al. (2015, 2017).

Before applying the MRDM and commonality analysis, we checked for multicollinearity among the final set of explanatory distances as recommended by Dormann et al. (2013). We conducted commonality analysis based on MRDM using the R code provided by Prunier et al. (2015), with 999 permutations to assess significance and 10,000 bootstraps to obtain confidence intervals of parameter estimates.

#### 2.3.4 Post hoc analysis of road type effects on genetic connectivity

**FIGURE 2:**
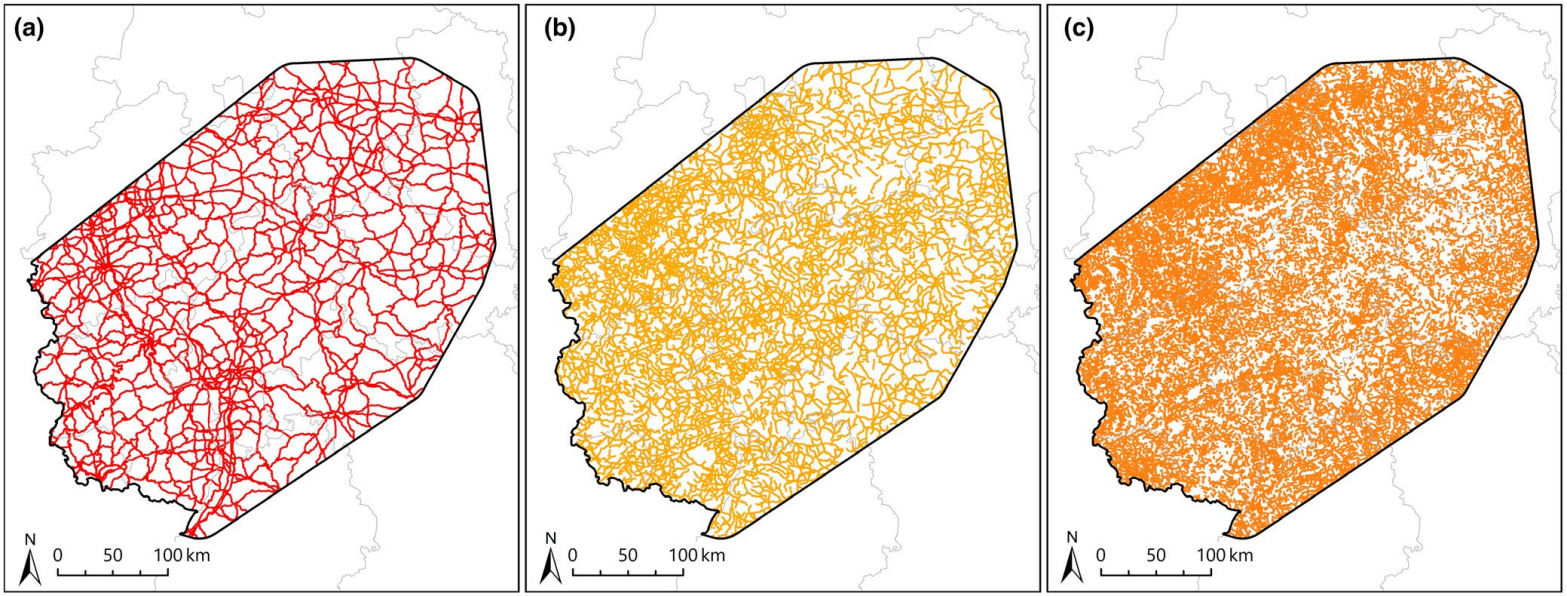
Road network in our study area separated by administrative responsibility (a = federal roads, b = state roads, c = county roads)

Results of the commonality analysis showed that road density plays a major role in determining the genetic structure of wildcats in Germany (see Section 3). To evaluate this effect in more detail, we conducted a *post hoc* analysis involving three different road types according to administrative responsibility (Figure 2): (a) federal highways and federal autobahn (“federal”), (b) rural roads under administration of the states (“state”), and (c) district and municipal roads (“county”). Road type data stem from a digital road layer provided by the Federal Agency for Cartography and Geodesy (2015). We calculated road density for each of these road types separately within a radius of 35 km and then followed the same analytical procedure described above; that is, we transformed density layers into resistance surfaces, estimated effective distances among individuals, used Mantel and partial Mantel tests to find the best representation for each road type, checked for multicollinearity, and used commonality analysis based on MRDM for final inferences.

## 3 RESULTS

### 3.1 Selection of effective distances of landscape variables

Simple Mantel tests were insignificant for the proportion of forest edge in a 1 km radius and for forest proportion within a radius of 5  and 10 km (Table S2). Furthermore, partial Mantel tests were insignificant for effective distances of distance to railroads, grassland proportion within a 5, 10, and 35 km radius, proportion of Global Urban Footprint within a 5 km radius, and proportion of forest interior within a 1 km radius (Table S2). Hence, we did not consider these variables for further analyses.

The best transformation for each remaining variable is reported in Table 2. We only considered variables with a partial Mantel *r* of >0.1 and correlations of <0.7 for final analyses (see Appendix S3.1, Table S3). Correlation between Global Urban Footprint and road density within a 35 km radius was >0.7, and due to a larger partial Mantel *r*, we decided to use road density in further analyses. The measures of Global Urban Footprint and road density are very similar and areas with large values of Global Urban Footprint also have high road density, so using just one of these variables is sufficient. For forests and agricultural land, the resistance representation was based on the proportion of these land cover types within 35 km radius. Effective distances based on these two variables were highly correlated (Mantel *r* = .83), but forest and agricultural land are ecologically clearly distinct, and hence, we kept both variables for further analyses.

This led to our final data set consisting of effective distances calculated from six landscape variables: (1) *road density* within a radius of 35 km (negative effect on genetic connectivity, i.e., higher road densities lead to higher resistance), (2) *proportion of forest* within a radius of 35 km (positive effect, i.e., higher densities of this land cover types decrease resistance), (3) *proportion of agricultural land* within a radius of 35 km (positive effect), (4) *distance to settlements* (positive effect), (5) *distance to Continuous Low Traffic Areas* (negative effect), and (6) *topographic slope* (negative effect). We included these six final variables in the same MRDM model, together with straight‐line distances to represent IBD. Statistically, including the two highly correlated variables *proportion of forest* and *proportion of agricultural lands* in a regression is not ideal (Dormann et al., 2013). Hence, we also created a new resistance layer by summing up the proportions of forest and agricultural lands within a 35 km radius and rescaled this layer to a resistance surface with values between 100 and 10,000, as described in the methods section, again assuming that both variables support gene flow. We recalculated effective distances based on this combined layer and included them as *forest–agricultural land* together with the other variables in an additional commonality analysis (Appendix S2.2).

### 3.2 Relative effects of landscape variables on genetic connectivity

**FIGURE 3:**
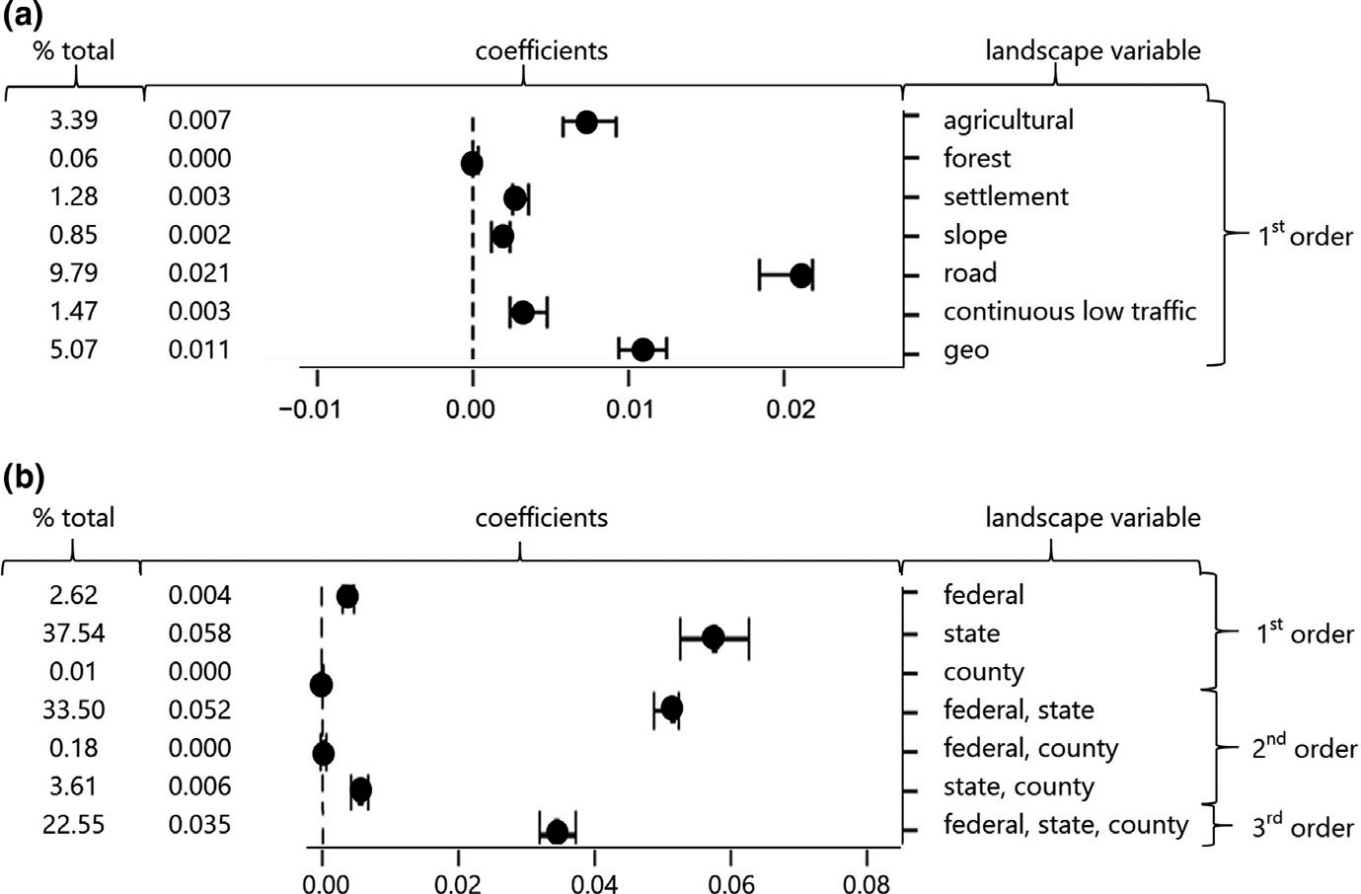
Results of the commonality analysis: coefficients with 95% confidence intervals and their contribution to the overall model *R* ^2^ (% total). Coefficients represent the percentage of variance explained by each set of landscape variables (a; only 1st^‐^order effects; see Figure S1 for all effects) and different road types (b). Forest = proportion of forest in 35 km radius, farm = proportion of agricultural land within 35 km radius, settlement = distance to settlements, road = road density within 35 km radius, continuous low traffic = distance to Continuous Low Traffic Areas, geo = straight‐line distance

The commonality analyses of the MRDM revealed pronounced differences in the relative effects of landscape variables on genetic connectivity (Table 3, Figure 3a). While all variables had a significant overall effect *T* (*p* ≤ .001, Table 3), road density had by far the largest unique effect *U* and contributed most strongly to the explained variation (Table 3, Figure 3a). Unique effects of all other variables were smaller than the unique effect of straight‐line distances, indicating that these other individual variables are less important than IBD for explaining gene flow in wildcats. However, the sum of contributions of the individual variables to the overall *R* ^2^ (.210) summed to >1 (Table 3), suggesting synergistic effects of variables on genetic connectivity.

**TABLE 3:** Results of the MRDM (weighted beta *β* and *p*‐value *p*) and commonality analysis (individual *U*, common *C,* and total *T* effect of each variable, and their contribution to the *R* ^2^ of the overall model) for the landscape variables (top) and *post hoc* analysis of road types (bottom)

Parameter	*β*	*p*	*U*	*C*	*T*	Proportion *R* ^2^
Landscape variables						
Proportion of agricultural land, 35 km radius	0.161	.001	0.007	0.014	0.021	.10
Proportion of forest, 35 km radius	0.022	.001	0.000	0.002	0.002	.01
Distance to settlements	0.075	.001	0.003	0.097	0.099	.47
Slope	0.066	.001	0.002	0.086	0.088	.42
Road density, 35 km radius	0.179	.001	0.021	0.096	0.117	.56
Distance to Continuous Low Traffic Areas	0.070	.001	0.003	0.067	0.070	.33
Straight‐line distance	0.196	.001	0.011	0.091	0.102	.49
Road types						
Federal	0.085	.001	0.004	0.087	0.091	.59
State	0.330	.001	0.058	0.092	0.150	.97
County	0.004	.356	0.000	0.041	0.041	.26

Note

Proportion *R* ^2^ represents the percentage of variance explained by each variable alone and in combination with other variables.

A particularly strong synergistic effect occurred between the proportion of forest and the proportions of agricultural land. Individually, these two variables are not very important for explain gene flow, and the unique effect of the proportion of forest was not different from zero (i.e., confidence intervals for 1st‐order effects overlapped zero; Figure 3a). However, in synergy, these two variables had an effect that exceeded the effect of IBD (see 2^nd^‐order effects in Figure S1). This synergistic effect of forests and agricultural areas was also confirmed by our additional commonality analysis, where we replaced the individual variables proportion of forest and proportion of agricultural land by effective distances calculated from the combined resistance surface. This new variable forest–agricultural land was clearly identified as the 2^nd^ most influential variable after road density, and had a unique effect larger than the effect of IBD (see Appendix S2.2 for details). Both commonality analyses also suggested topographic slope to influence gene flow in wildcats, though the effect of this variable is only apparent in synergy with straight‐line distance, as indicated by the substantial 2^nd^‐order effects (Figures S1 and S2).

### 3.3 Relative effects of different road type densities on genetic connectivity

The densities of federal, state, and county roads all had a significant influence on the genetic structure according to Mantel and partial Mantel tests (*p* = .001, Appendix S2.3, Table S7). Correlations between these three variables ranged between 0.45 and 0.65 (Table S8); thus, we included all of them in the commonality analysis. The MRDM of the commonality analysis showed that county road density has no significant effect on wildcat genetic structure and that federal road density does not affect genetic structure by itself (*U* = 0.004; Table 3, Figure 3b), but rather in combination with state roads (combined effect *C* in Table 3). State road density exhibited by far the largest unique effect, and it had a total contribution of 97% to the overall model *R* ^2^ (.154; Table 3).

## 4 DISCUSSION

Maintaining or improving functional landscape connectivity is particularly important in highly fragmented landscapes, where sufficient amounts of dispersal and genetic exchange are critical to prevent species extinction and loss of genetic diversity (Haddad et al., 2015; Hanski, 2011). Planning of connectivity conservation in such landscapes should ideally be based on empirical data on dispersal movements and gene flow (Cushman et al., 2013; Epps et al., 2007; Zeller et al., 2018). Here, we objectively evaluated landscape effects on genetic connectivity in the European wildcat, which is a prominent species used to plan defragmentation efforts in Germany and other European countries. Using an individual‐based landscape genetic framework and multivariate inferential statistics, we show that genetic structure in the German core distribution of wildcats is influenced by IBD and six synergistically interacting landscape variables. In detail, road density, the proportions of forest and agricultural land, the distances to Continuous Low Traffic Areas, and settlements and topographic slope best explain spatial genetic structure. Of these variables, road density clearly has the strongest effect on gene flow and this effect was largely explained by the density of state roads. These results generally corroborate previous findings and assumptions about landscape effects on wildcat dispersal movements (Götz et al., 2018; Jerosch et al., 2017; Klar et al., 2008), but also refine our understanding of the species in a way that has great relevance for practical connectivity conservation.

### 4.1 Landscape impacts on genetic connectivity in wildcats

Our study shows that several tested variables have effects on wildcat genetic connectivity across the study region (Figure 1). Given the large extend of the area, it is not surprising that we detected a significant IBD pattern, as gene flow in this medium‐sized mammal can be expected to decrease across large spatial distances. Previous genetic studies on European wildcats and other felids have also detected significant IBD (Balkenhol et al., 2014; Hartmann et al., 2013). Previous studies have already shown the dependence of wildcats on forested areas for both residency and movement (Anile et al., 2019; Klar et al., 2008). According to our results, forested areas by themselves do not appear to facilitate gene flow, but there was a strong synergistic effect with agricultural land. Recent studies have suggested that wildcats can utilize agricultural fields, provided that they are structurally diverse and offer sufficient shelter, for example in form of hedgerows or shrubs (Götz et al., 2018; Jerosch et al., 2017; Lozano, 2015). Thus, our results suggest that it is the mix of forests and agricultural land that is most beneficial for wildcat gene flow. Unfortunately, while our findings suggest that genetic connectivity increases with increasing amounts of forest and agricultural land, the spatial resolution of our landscape data prevents us from quantifying the effects of fine‐scale habitat structures within these areas. It is possible that certain agricultural fields actually provide high resistance to dispersing wildcats, for example, when vegetative cover is completely removed from large fields via harvesting. Thus, future research is necessary to clarify whether the positive association with gene flow is limited to structurally diverse agricultural land.

Steep topographic slopes also impeded gene flow in wildcats, though mostly in synergy with straight‐line distances. This could indicate that steep slopes only provide high resistance to wildcats if they occur across long spatial distances. For example, Monterroso et al. (2009) showed that radio‐tracked wildcats in a National Park in Portugal actually preferred areas with higher slopes to avoid anthropogenic disturbance in a topographically rugged landscape. As our study area is much larger and topographically more diverse with higher and steeper mountains but also flat areas, it seems plausible that steep slopes in our study area provide higher resistance to wildcat dispersal movements than flat areas.

Various previous studies have shown that wildcats avoid human settlements and proximity to such settlements (Birlenbach & Klar, 2009; Klar et al., 2008; Oliveira et al., 2018). Consequently, our results show that areas close to human settlements provide a significant resistance to gene flow, even though it is less influential than IBD. The variable had its greatest effect in synergy with other variables (Figures S1 and S2), which highlights that it is not just proximity to settlements that provides high resistance in some areas, but also the presence of other landscape variables, such as high road densities or low amounts of agricultural land or forests. The same was found for the distance to Continuous Low Traffic Areas: The effect of this variable was only influential in conjunction with other variables, indicating that proximity to Continuous Low Traffic Areas is indeed beneficial, but only when other landscape characteristics (e.g., high amounts of forests or agricultural land) facilitate genetic exchange.

Several other variables we tested led to low correlations with genetic distances, including railways, rivers, Global Urban Footprint, grasslands, and two complementary indices of forest fragmentation. While the linear features railways and rivers might still have local, rather than landscape‐wide effects, the other variables do not appear to impact wildcat gene flow in our study area. The habitat suitability model we used had no significant effect on genetic connectivity of wildcat. This shows that habitat suitability explaining the occurrence of wildcats is not the same as landscape resistance for dispersal movements. Specifically, various studies have shown that animals may still move through habitat with low suitability during dispersal and that predicting movement and gene flow from habitat suitability models is not always possible (Abrahms et al., 2017; Keeley et al., 2017; Mateo‐Sánchez et al., 2015b).

Overall, the most important landscape variable affecting genetic connectivity in our study was road density. This variable largely impeded gene flow with by far the largest unique effect and was always significant when combined with other variables, which is why we added an additional analysis on effects of different road types.

### 4.2 Road type impacts on genetic connectivity in wildcats

Our analyses revealed a strong impact of road densities on gene flow in wildcats: While the effect of county road density is negligible, state road density is most important, followed by federal roads. Landscape connectivity decreases as a consequence of roads in a variety of species (Balkenhol & Waits, 2009; Holderegger & Di Giulio, 2010; van der Ree et al., 2015), for example due to hindered dispersal and patch reachability (Klar et al., 2006; Kramer‐Schadt et al., 2004; Zimmermann et al., 2007), but also due to direct road mortality (Fahrig & Rytwinski, 2009; Kramer‐Schadt et al., 2004). Previous research has also shown that the probability of successfully crossing roads depends on their width, traffic volumes, and vehicle speeds (Alexander et al., 2005; Clevenger et al., 2003; Gagnon et al., 2007; Huijser & McGowen, 2010; Meisingset et al., 2014; Yanes et al., 1995). Data on these factors are not available for the various roads in our study area; hence, we are not able to explicitly test for the impact of road characteristics on genetic structure. However, the typical speed limit on federal roads in Germany is higher compared with state and county roads, and federal roads are usually wider and receive substantially more traffic than other roads. For example, monitored sections of federal roads in the state of Hesse were used by ca. 76,307 vehicles in a typical 24‐hr period in 2015, while monitored sections of state roads were used by only ca. 3,161 vehicles per day (Hessen Mobil, 2015). Hartmann et al. (2013) showed that a major, 6‐lane highway with >100,000 vehicles per day was a severe impediment to wildcat gene flow in central‐western Germany, illustrating that major federal roads can indeed act as local barriers for wildcats. So, why do our results suggest that state roads have a much stronger impact on wildcat gene flow than federal roads? We believe that this is explained by the high number and widespread distribution of state roads in Germany, where state roads are much more abundant than federal roads (Figure 2a,b). This indicates that state roads could be a more common and *landscape‐wide* source of mortality for wildcats, even if federal roads exert a higher *local* barrier effect on gene flow.

Especially in densely populated countries with large amounts of roads, mortality risk of wildlife can be high (Meijer et al., 2018), and for European wildcats in Germany, roads are assumed to be the main source of mortality (Echle et al., 2018; Pott‐Dörfer & Raimer, 2007; Simon & Raimer, 2005; Steyer et al., 2016). Hence, it seems reasonable that resistance to gene flow increases with higher state road densities, simply because every crossing of a state road is associated with a certain mortality risk. In contrast, the even more abundant county roads (Figure 2c) did not have a significant impact on genetic connectivity in our study system, probably because these roads are usually narrow infrastructures with lowest traffic volumes and vehicle speeds. Thus, the landscape‐wide effects of different road types on genetic connectivity appear to be shaped not only by road characteristics, but also by the relative abundance of different roads. More generally, our findings suggest that many landscape variables with small or intermediate barrier effect can have a more pronounced landscape‐wide impact on gene flow than few landscape variables with a large barrier effect.

### 4.3 Conservation implications

Our findings have important implications for ongoing activities to increase landscape connectivity for wildcats across Germany. First, connectivity efforts for the wildcat in Germany usually do not consider agricultural land as potential low‐resistance areas. Agricultural land was not included in the original habitat selection study that several of the current wildcat conservation projects are based on (i.e., Klar et al., 2008), most likely because the wildcat has traditionally been considered a forest‐dependent species in Germany. However, more recent studies suggest that the species can use agricultural land, as long as these areas provide ample structural diversity and associated cover (Götz et al., 2018; Jerosch et al., 2017, 2018). Our results similarly suggest that genetic connectivity is high across agricultural lands, indicating that agricultural land might offer an underestimated potential to support successful wildcat dispersal movements. Thus, we encourage future studies to investigate the exact impact of different agricultural fields on wildcat dispersal and mating movements, and to evaluate under which circumstances agricultural land represents a conduit for gene flow, and when they represent an impediment.

Second, currently used corridor networks for the species in Germany are based on predicted wildcat movement paths that do not account for the potential effects of road density on realized connectivity. Klar et al. (2008) showed that radio‐collared wildcats significantly avoided close proximity to roads within their home ranges, and this effect is explicitly considered in a resistance model used to guide local re‐connection efforts for the species (e.g., Klar et al., 2012). However, *distance to roads* did not explain range‐wide genetic structure in our study, while we clearly identified *state road density* as a major determinant of wildcat genetic connectivity in Germany. Hence, we suggest that including the latter variable is crucial to identify areas within the country that have particularly high resistance to gene flow, which can help to refine predicted movements paths across large spatial extents and provide important additional detail for corridor design and mitigation measures.

More broadly, the fact that state roads were the most important predictor of landscape‐wide genetic connectivity in our study has interesting implications for connectivity conservation in general. Roads have been confirmed as complete or partial barriers for a variety of species around the globe (Balkenhol & Waits, 2009; Holderegger & Di Giulio, 2010), and crossing structures, such as wildlife over‐ or underpasses, are often used to mitigate such effects (Smith et al., 2015). However, in many cases, these structures are constructed across major roads, such as federal highways, as they presumably present the most severe impediments to animal movements.

While decreasing the local barrier effect of major federal roads is certainly beneficial for affected wildlife species, our results highlight that also other roads can actually resemble severe impediments to landscape‐wide connectivity, most likely because they are highly abundant and pervasive. Thus, road mitigation should not solely focus on local effects of the most prominent transportation infrastructures, but should additionally identify the type of roads that have the strongest influence on landscape‐wide connectivity and then consider crossing structures across these roads as an important part of connectivity conservation. Moreover, since it is highly unlikely that mitigation measures will ever provide safe passage for wildlife across all currently problematic roads, we emphasize that future efforts should try to strengthen local and national public transport to decrease overall traffic volumes, and to minimize landscape‐wide road construction as much as possible, especially in areas that are already characterized by a dense network of transportation infrastructures.

### 4.4 Conclusions and future research needs

Our study demonstrates how a large‐scale, multivariate landscape genetic analysis can help to refine our understanding of functional connectivity in a focal species for conservation. Future studies should evaluate how the existing corridor network for the wildcat changes when including agricultural land and (state) road densities as additional variables in the underlying resistance model. Furthermore, we suggest to validate whether the strong effect of state road density observed in our study is really due to high road mortality, as we suspect, or also due to a behavioral avoidance of road crossings. For example, Fletcher et al. (2019) recently introduced a method based on spatial absorbing Markov chains for distinguishing the relative impacts of behavioral movement barriers versus mortality on realized connectivity. Within the same context, the role of traffic volumes, vehicle speed, and road width for connectivity and gene flow in wildcats should be evaluated to develop guidelines on the most promising mitigation measures (e.g., fencing to reduce mortality vs. crossing structures to reduce behavioral avoidance; see Klar et al., 2009; Spanowicz et al., 2020). Finally, landscape connectivity for the wildcat needs to be compared with that of other wildlife species in Germany. For example, red deer (*Cervus elaphus*), Eurasian lynx (*Lynx lynx*), Gray wolf (*Canis lupus*), and Eurasian otter (*Lutra lutra*) have also been used as focal species for nationwide connectivity planning in the country (Herrmann et al., 2007). Since available empirical data on movements and gene flow vary widely across these species, expert opinion is commonly used to predict most likely movement paths and identify locations for mitigation measures. We suggest that large‐scale landscape genetic studies for these different species could shed additional light on the factors that facilitate or hinder their successfully dispersal movements, clarify how redundant or complementary these species are for connectivity planning, and help to create an objective, evidence‐based connectivity plan for multiple species across Germany and other European countries.

## CONFLICT OF INTEREST

The authors declare no conflict of interest.

## AUTHORS CONTRIBUTION

**Katharina Westekemper:** Conceptualization (equal); Data curation (equal); Formal analysis (equal); Funding acquisition (equal); Project administration (lead); Software (equal); Visualization (lead); Writing‐original draft (lead). **Annika Tiesmeyer:** Data curation (lead); Resources (equal); Writing‐original draft (supporting). **Katharina Steyer:** Data curation (equal); Resources (supporting); Writing‐original draft (supporting). **Carsten Nowak:** Supervision (equal); Writing‐original draft (supporting). **Johannes Signer:** Formal analysis (equal); Methodology (equal); Software (equal); Writing‐original draft (supporting). **Niko Balkenhol:** Conceptualization (equal); Funding acquisition (lead); Methodology (equal); Project administration (equal); Software (equal); Supervision (lead); Writing‐original draft (equal).

## Supporting information

Figure S1

Figure S2

Supplementary Material

## Data Availability

Data on pairwise genetic and geographic distances as well as effective distances based on resistance surfaces of various landscape variables in different transformations have been archived at Dryad: https://doi.org/10.5061/dryad.6wwpzgmvq. [Correction added on 3 June 2021, after first online publication: The private link to access the data temporarily has been removed in this version.]
