# Supplementary figures and images for: Do all roads lead to resistance? State road density is the main impediment to gene flow in a flagship species inhabiting a severely fragmented anthropogenic landscape
Source: Ecol Evol. 2021 May 6;11(13):8528–41. doi: 10.1002/ece3.7635 (PMC8258205; doi:10.1002/ece3.7635)

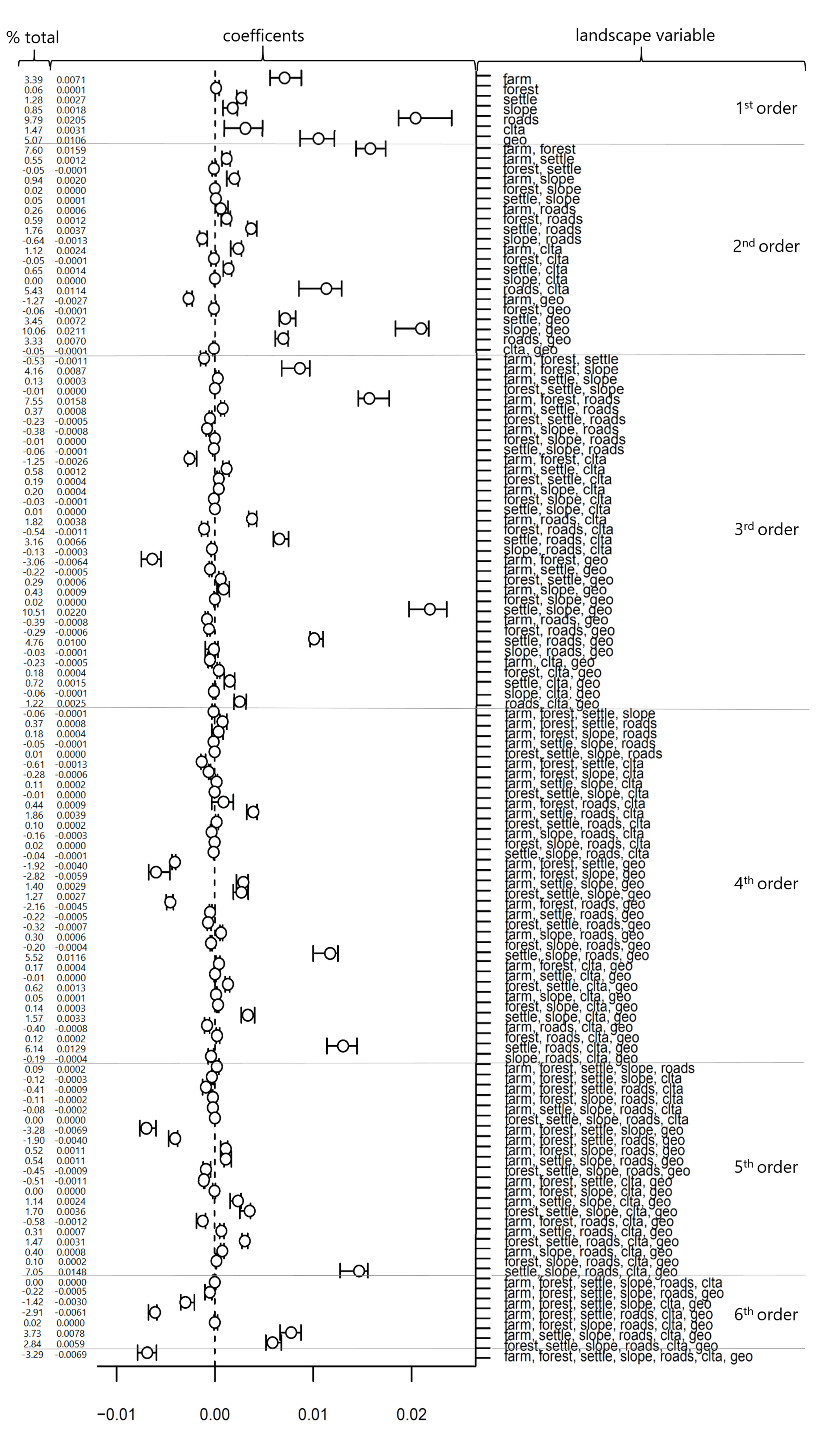

Supplement: Supplementary file 1 — Figure S1 [file ECE3-11-8528-s001.jpg]

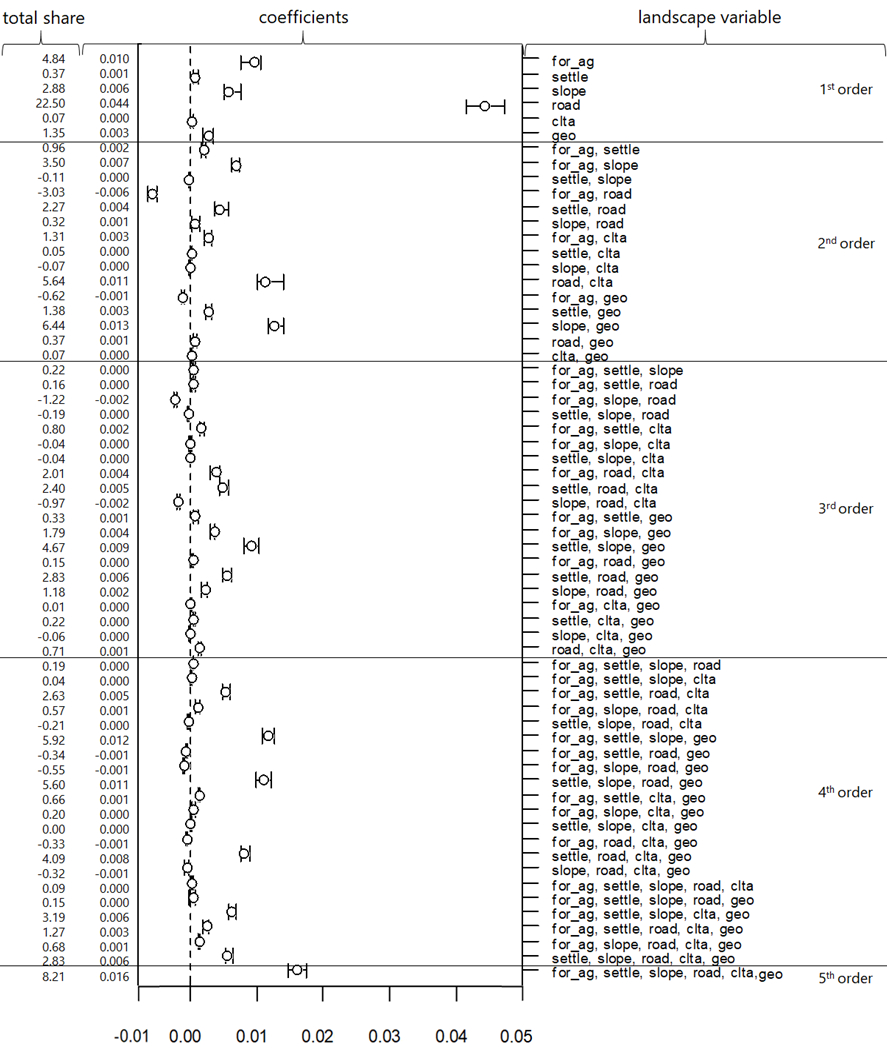

Supplement: Supplementary file 2 — Figure S2 [file ECE3-11-8528-s002.jpg]
