# Supplementary material for: Do all roads lead to resistance? State road density is the main impediment to gene flow in a flagship species inhabiting a severely fragmented anthropogenic landscape
Source: Ecol Evol. 2021 May 6;11(13):8528–41. doi: 10.1002/ece3.7635 (PMC8258205; doi:10.1002/ece3.7635)
Supplement: Supplementary file 3 — Supplementary Material [file ECE3-11-8528-s003.docx]

## Appendix

### Appendix 1 - Population genetics

Table S1. Population genetic parameter for the entire dataset. Number of genotypes (N), number of alleles (NoA), allelic richness (Ar) expected (H_e_) and observed heterozygosity (H_o_).

| **N** | **NoA** | **A_r_** | **H_e_** | **H_o_** |
| --- | --- | --- | --- | --- |
| 975 | 146 | 5.62 | 0.64 | 0.61 |

### Appendix 2

#### Appendix 2.1 Landscape genetics

Table S2. Results of simple and partial Mantel tests of effective distances based on all resistance surfaces used. Best transformation of each landscape variable is indicated as underlined partial Mantel r, effective distances of landscape variables used for commonality analyses are shown in bold. Best transformations not used for final commonality analyses led to partial Mantel r < 0.01. Global Urban Footprint was not used in final analyses due to its’ high correlation with road density (see Table S3 and main text for details).

| **Landscape variable** | **Mantel *r*** | ***p*** | **partial Mantel *r*** | ***p*** |
| --- | --- | --- | --- | --- |
| Agricultural land, 5 km | -0.074 | 0.001 | -0.165 | 0.001 |
| Agricultural land, 10 km | -0.097 | 0.001 | -0.193 | 0.001 |
| **Agricultural land, 35 km** | **0.144** | **0.001** | **0.254** | **0.001** |
| Agricultural land, distance | 0.216 | 0.001 | -0.014 | 0.484 |
| **Continuous Low Traffic Areas, distance** | **0.264** | **0.001** | **0.176** | **0.001** |
| Forest edge, 1 km | -0.030 | 0.138 | -0.174 | 0.001 |
| Forest interior, 1 km | 0.103 | 0.001 | -0.030 | 0.135 |
| Forest, 5 km | -0.003 | 0.880 | -0.104 | 0.001 |
| Forest, 10 km | -0.032 | 0.108 | -0.182 | 0.001 |
| **Forest, 35 km** | **0.048** | **0.022** | **0.200** | **0.001** |
| Forest, distance | 0.182 | 0.001 | -0.091 | 0.001 |
| Grassland, 5 km | 0.234 | 0.001 | 0.014 | 0.476 |
| Grassland, 10 km | 0.185 | 0.001 | 0.032 | 0.095 |
| Grassland, 35 km | 0.150 | 0.001 | 0.037 | 0.070 |
| Grassland, distance | 0.175 | 0.001 | 0.073 | 0.001 |
| Global Urban Footprint, 5 km | 0.129 | 0.001 | 0.027 | 0.153 |
| Global Urban Footprint, 10 km | 0.313 | 0.001 | 0.146 | 0.001 |
| Global Urban Footprint, 35 km | 0.246 | 0.001 | 0.163 | 0.001 |
| Habitat suitability model | 0.278 | 0.001 | 0.056 | 0.009 |
| **Isolation by distance** | 0.327 | 0.001 | - | - |
| Railroad, distance | 0.202 | 0.001 | 0.036 | 0.081 |
| River, distance | 0.112 | 0.001 | 0.040 | 0.038 |
| Roads, 5 km | 0.247 | 0.001 | 0.126 | 0.001 |
| Roads, 10 km | 0.354 | 0.001 | 0.249 | 0.001 |
| **Roads, 35 km** | **0.342** | **0.001** | **0.269** | **0.001** |
| Roads, distance | 0.329 | 0.001 | 0.158 | 0.001 |
| **Settlement, distance** | 0.315 | 0.001 | 0.145 | 0.001 |
| **Slope** | 0.296 | 0.001 | 0.104 | 0.001 |

Table S3. Correlation between effective distances of selected landscape variables.

|  | **Forest, 35 km** | **Global Urban Footprint, 35 km** | **Settlement, distance** | **Slope** | **Roads, 35 km** | **Continuous Low Traffic Areas, distance** |
| --- | --- | --- | --- | --- | --- | --- |
| **Agricultural land, 35 km** | 0.827 | -0.023 | -0.128 | 0.054 | 0.144 | -0.011 |
| **Forest, 35 km** |  | -0.165 | -0.295 | -0.073 | 0.066 | -0.201 |
| **Global Urban Footprint, 35 km** |  |  | 0.384 | 0.125 | 0.744 | 0.494 |
| **Settlement, distance** |  |  |  | 0.473 | 0.411 | 0.454 |
| **Slope** |  |  |  |  | 0.188 | 0.214 |
| **Roads, 35 km** |  |  |  |  |  | 0.488 |


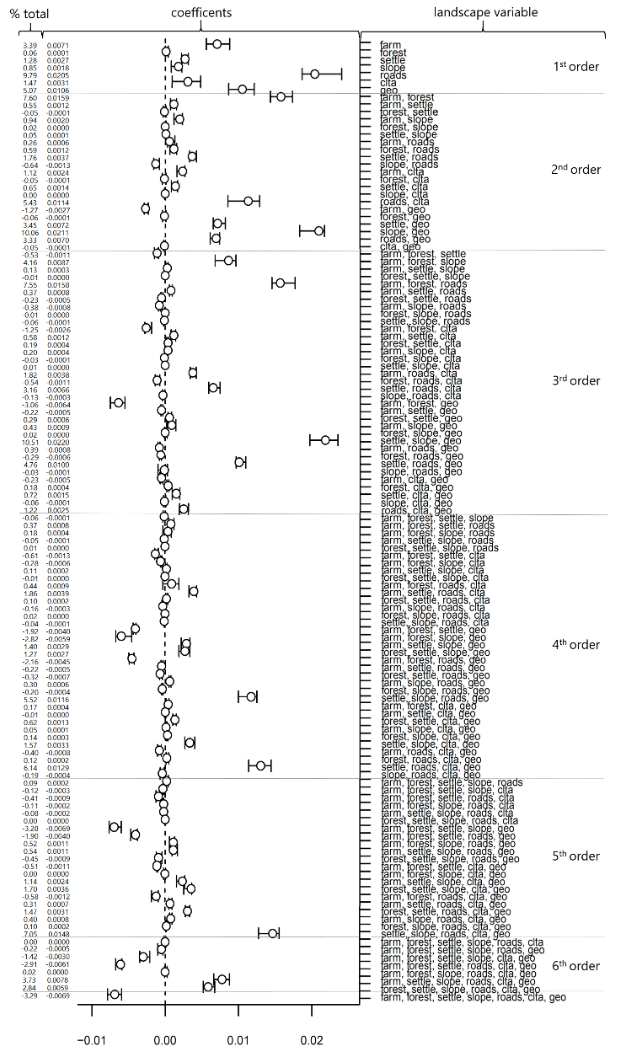


Figure S1. Complete results of the commonality analysis: coefficients with 95 % confidence intervals and their contribution to the overall model R² (% total). Coefficients represent the percentage of variance explained by each set of landscape variables (Farm = proportion of agricultural land within 35 km radius, forest = proportion of forest within 35 km radius, settle = distance to settlements, road = road density within a 35 km radius, clta = distance to Continuous Low Traffic Areas, geo = straight-line distance).

#### Appendix 2.2 - Merged forest and agricultural land

Table S4. Results of simple and partial Mantel tests of effective distances based on proportion of forest-agricultural land within 35 km radius.

| **Landscape variable** | **Mantel *r*** | ***p*** | **partial Mantel *r*** | ***p*** |
| --- | --- | --- | --- | --- |
| Forest-agricultural land, 35 km | 0.257 | 0.001 | 0.169 | 0.001 |

Table S5. Correlation between effective distances of best landscape variable transformation and effective distances of proportion of forest-agricultural land within 35 km radius.

| **Landscape variable** | **Forest-agricultural land, 35 km** |
| --- | --- |
| Grassland, distance | 0.241 |
| Global Urban Footprint, 35 km | 0.177 |
| Habitat suitability model | 0.347 |
| Settlement, distance | 0.430 |
| River, distance | 0.056 |
| Slope | 0.422 |
| Roads, 35 km | 0.135 |
| Continuous Low Traffic Areas, distance | 0.442 |

Table S6. Results of the MRDM (weighted beta *β*, and p-value *p*) and commonality analysis (individual *U*, common *C* and total *T* effect of each variable, and their contribution to the R² of the overall model) for the landscape variables. *Proportion R²* represents the percentage of variance explained by each variable alone and in combination with other variables.

| **Landscape variable** | ***β*** | ***p*** | ***U*** | ***C*** | ***T*** | **Proportion *R²*** |
| --- | --- | --- | --- | --- | --- | --- |
| Road density in 35 km radius | 0.253 | 0.001 | 0.044 | 0.073 | 0.117 | 0.60 |
| Proportion of forest-agricultural land in 35 km radius | 0.122 | 0.001 | 0.010 | 0.056 | 0.066 | 0.34 |
| Slope | 0.113 | 0.001 | 0.006 | 0.082 | 0.088 | 0.45 |
| Straight-line distance | 0.088 | 0.001 | 0.003 | 0.099 | 0.102 | 0.52 |
| Distance to settlements | 0.040 | 0.001 | 0.001 | 0.099 | 0.099 | 0.51 |
| Distance to Continuous Low Traffic Areas | 0.016 | 0.002 | 0.000 | 0.070 | 0.070 | 0.36 |


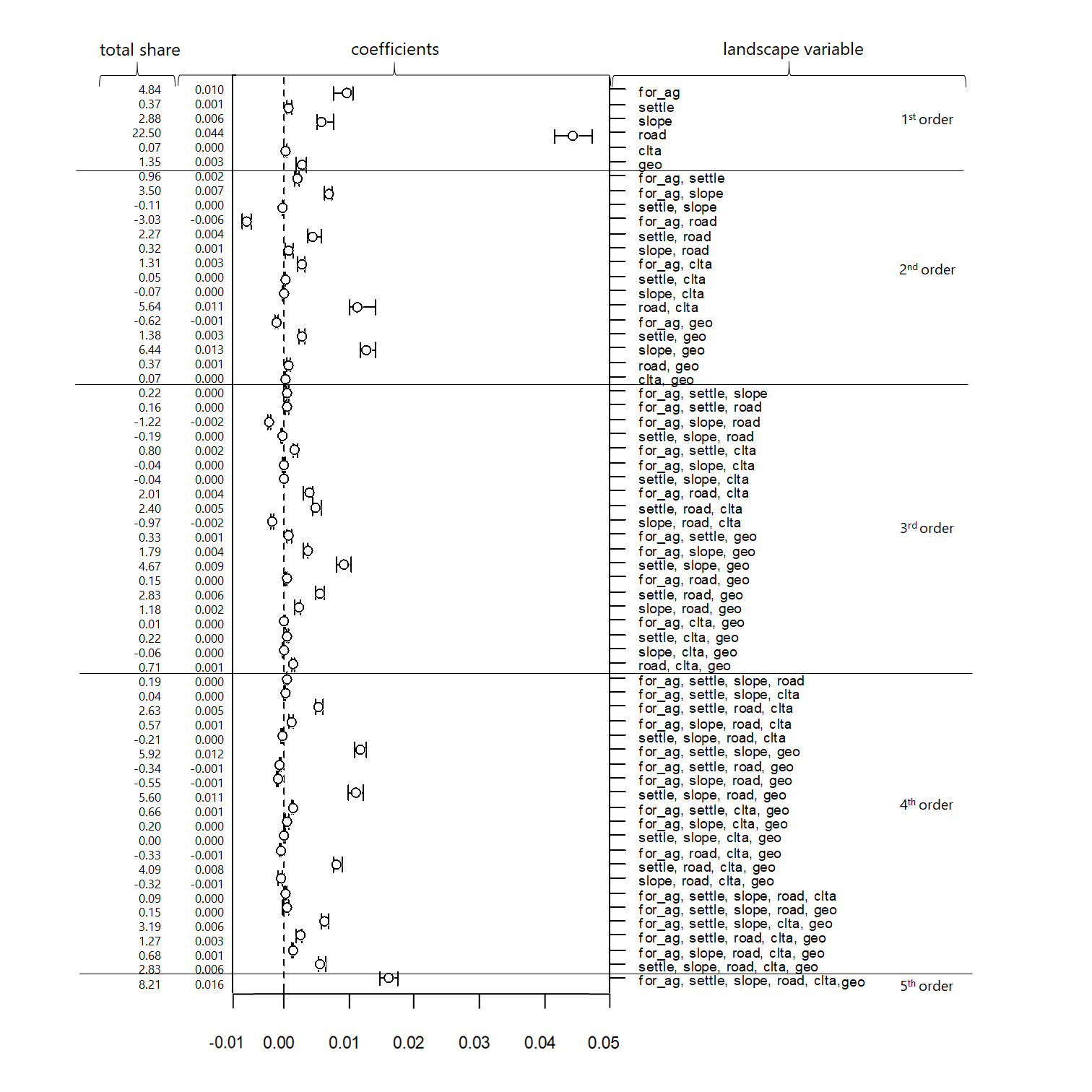


Figure S2. Results of the commonality analysis: coefficients with 95 % confidence intervals and their contribution to the overall model R² (% total). Coefficients represent the percentage of variance explained by each set of landscape variables (For_ag = proportion of forest-agricultural land within 35 km radius, settle = distance to settlements, road = road density within a 35 km radius, clta = distance to Continuous Low Traffic Areas, geo = straight-line distance).

#### Appendix 2.3 - Road Types

Table S7. Results of simple and partial Mantel tests of effective distances based on road types separated by administrative responsibility.

| **Road type** | **Mantel *r*** | ***p*** | **partial Mantel *r*** | ***p*** |
| --- | --- | --- | --- | --- |
| Federal | 0.301 | 0.001 | 0.239 | 0.001 |
| State | 0.387 | 0.001 | 0.323 | 0.001 |
| County | 0.201 | 0.001 | 0.088 | 0.001 |

Table S8. Correlation of effective densities based on road types separated by administrative responsibility.

|  | **State** | **County** |
| --- | --- | --- |
| **Federal** | 0.649 | 0.447 |
| **State** |  | 0.482 |
